# O27 Recurrent severe uveitis treated with secukinumab following anti-TNF induced cutaneous vasculitis in a patient with axial spondyloarthritis

**DOI:** 10.1093/rap/rkab067.026

**Published:** 2021-10-19

**Authors:** Antoni Chan, Sarah Sacks

**Affiliations:** Department of Rheumatology, Royal Berkshire NHS Foundation Trust, Reading, United Kingdom

## Abstract

**Case report - Introduction:**

Uveitis is the most common extra-articular manifestation in axial spondyloarthritis (axSpa) and can affect up to a third of patients. It usually presents acutely and unilaterally in the anterior aspect as iritis. It is treated with topical steroids, mydriatics and immunosuppresants. Anti-TNF has been used successfully to treat uveitis in the context of axSpa and is the cornerstone of long-term control of recurrent uveitis. In severe cases, surgery in the form of vitrectomy is done. In axSpa patients who have failure or adverse effects from anti-TNF, alternative treatments are required to prevent the complications of recurrent uveitis and visual loss.

**Case report - Case description:**

A 68-year-old female patient with axSpA presented with recurrent anterior uveitis in the left eye. She had radiographic axSpA (ankylosing spondylitis) for 6 years and was HLA-B27 positive. Her past medical history included hypertension which was treated with amlodipine and ramipril. She was treated with adalimumab for 3 months for her axSpa and this was stopped due to intolerance. She was then switch to etanercept which controlled her axial and peripheral joints symptoms but she developed recurrent anterior uveitis. She remained on etanercept for 2 years. The uveitis was treated with Maxidex, dorzolamide and brimonidine eye drops. Despite this, her uveitis progressed and closed angle glaucoma and cataract. She was then switched to golimumab in order to gain control of her uveitis. She developed cutaneous vasculitis after starting treatment. She presented with a widespread vasculitic rash over her calves with areas of central necrosis. Her investigations revealed a WCC 15.04, plts 456, neut 9.17, eosin 0.30, ESR 84, CRP 45, U&E normal, ALP 178, ALT 36. Urine dip was negative for protein/blood/leuc/nitrites. Chest X-ray showed mild bronchial wall thickening in the right lower zone with a few ill-defined small opacites of indeterminate significance. Blood cultures x3 were negative. Echocardiogram was normal with no vegetations. CT thorax was normal. ANA 1:160, ENA negative, RF/anti-CCP negative, ANCA negative, C3 1.88, C4 0.38. She was commenced on prednisolone 20mg once a day weaning regime once infection was excluded. Her anti-TNF golimumab was stopped. She made a dramatic improvement in her cutaneous vasculitis after withdrawal of anti-TNF but developed attacks of uveitis in the left eye. This was treated trabeculectomy, intravitreal injection of Ozurdex and a YAG capsulotomy. She was commenced on secukinumab to treat her active axSpa and this has stabilised her uveitis.

**Case report - Discussion:**

Anti-TNF biologic drugs are effective in treating axial, peripheral joint as well as extra-articular manifestations (EAMs) in axSpa. Uveitis is the commonest EAM in axSpa and responds very well to anti-TNF drugs. The presence of HLA-B27 positivity as in our case, predisposes the patient to uveitis and EAMs. Uveitis may lead to complications such as glaucoma, cataracts, cystoid macular oedema, detached retina and posterior synechiae. In our case, the patient had the first three complications as a result of recurrent uveitis and withdrawal of anti-TNF due to adverse events. She required surgery include trabeculectomy, intra-vitreal injection and capusulotomy. Ongoing systemic treatment is required in her to avoid any further loss of vision.

In a third of axSpa patients there may be failure or adverse reaction to anti-TNF. In patients who have failed anti-TNF they may be switched to another anti-TNF such as in our case. In patients with adverse reactions to anti-TNF, the choice is to either re-challenge with anti-TNF or switch to another drug with a different mode of action. For axSpa, the options include anti-IL17 and JAK inhibitors. Adverse reactions to anti-TNF include cutaneous manifestations such as cutaneous vasculitis as in our case, cutaneous lupus, psoriasis, hidradenitis suppurativa, lichen planus, vitiligo and alopecia areata. Having stopped anti-TNF due to the cutaneous vasculitis, we switched her to secukinumab for control of her axSpa. This also resulted in improved control of her uveitis with reduced frequency and severity of uveitis.

In three phase 3 RCTs (SHIELD, INSURE, ENDURE) aiming to determine efficacy of secukinumab in non-infectious uveitis (Behcet's and non-Behcet's) against placebo, there was no significant differences in uveitis recurrence between secukinumab treatment groups and placebo groups in any study (primary end-point). More studies are required to assess the efficacy of IL-17 inhibitors in treating and preventing uveitis in axSpa.

**Case report - Key learning points:**

Uveitis is the most common extra-articular manifestation (EAM) in axSpa. Early assessment and treatment are required to prevent complications such as visual loss. Close liaison and working with ophthalmologists is essential in order to improve patient outcome in axSpa. In severe cases of uveitis with complications, surgery may be required to prevent visual loss.

Anti-TNF drugs are very effective in treating axSpa and are also beneficial in treating uveitis. A third of axSpa patients may have failure or adverse reaction to anti-TNF. The adverse event may include cutaneous manifestations such as vasculitis. This may necessitate withdrawal of anti-TNF.

In patients who are not able to take anti-TNF, suitable alternatives such as anti-IL-17 and JAK inhibitors are used to treat axSpa. The impact of anti-IL17 and JAK inhibitors on uveitis in axSpa is yet to be determined. In our case, anti-IL17 secukinumab was shown to be effective in reducing the recurrence of uveitis. This requires further research to study its efficacy in treating and preventing uveitis in axSpa.

